# Higher testosterone is associated with open-angle glaucoma in women: a genetic predisposition?

**DOI:** 10.1186/s13293-023-00512-z

**Published:** 2023-05-09

**Authors:** Joëlle E. Vergroesen, Adem Kaynak, Elif Aribas, Maryam Kavousi, Joyce B. J. van Meurs, Caroline C. W. Klaver, Wishal D. Ramdas

**Affiliations:** 1 Department of Ophthalmology, Erasmus MC University Medical Center, PO Box 2040, 3000 CA Rotterdam, The Netherlands; 2 Department of Epidemiology, Erasmus MC University Medical Center, PO Box 2040, 3000 CA Rotterdam, The Netherlands; 3 Department of Internal Medicine, Erasmus MC University Medical Center, PO Box 2040, 3000 CA Rotterdam, The Netherlands; 4 Department of Ophthalmology, Radboud University Medical Center, PO Box 9101, 6500 HB Nijmegen, The Netherlands; 5 Institute of Molecular and Clinical Ophthalmology, University of Basel, CH-4031 Basel, Switzerland

**Keywords:** Open-angle glaucoma, Intraocular pressure, Testosterone, Hormones, Sex-differences

## Abstract

**Background:**

Testosterone may be a possible modifiable risk factor for open-angle glaucoma (OAG) and intraocular pressure (IOP), but evidence has been scarce and conflicting. In this study we evaluated the association of testosterone and its genetic predisposition with incident (i) OAG, IOP, retinal nerve fiber layer (RNFL), and ganglion cell-inner plexiform layer (GCL +).

**Methods:**

Participants aged 45–100 years were derived from the prospective, population-based Rotterdam Study. Ophthalmic examinations and serum testosterone measurements (including bioavailable and free testosterone) were performed from 1991 onwards. Follow-up took place every 4–5 years. A total of 187 out of 7898 participants were diagnosed with incident (i) OAG during follow-up. Genotyping was performed in 165 glaucoma cases and 6708 controls. We calculated sex-specific weighted genetic risk scores (GRS) for total and bioavailable testosterone. Associations with iOAG were analyzed using multivariable logistic regression. Associations with IOP, RNFL, and GCL + were analyzed with multivariable linear regression. Analyses were stratified on sex and adjusted for at least age, body mass index, and follow-up duration.

**Results:**

In men, testosterone was not associated with iOAG. However, the GRS for higher total testosterone was associated with an increased iOAG risk (odds ratio [OR] with 95% confidence interval [95% CI]: 2.48 [1.18; 5.22], per unit). In women, higher values of bioavailable testosterone (2.05 [1.00; 4.18] per nmol/L) and free testosterone (1.79 [1.00; 3.20] per ng/dL) were significantly associated with increased risk of iOAG. Moreover, the GRS for higher bioavailable testosterone was associated with an increased iOAG risk (2.48 [1.09; 5.65], per unit). Higher bioavailable and free testosterone were adversely associated with IOP (0.58 [0.05; 1.10] per nmol/L and 0.47 [0.04; 0.90] per ng/dL). Higher total testosterone was inversely associated with peripapillary RNFL and GCL + (Beta [95% CI]: − 3.54 [− 7.02; − 0.06] per nmol/L and − 2.18 [− 4.11; − 0.25] per nmol/L, respectively).

**Conclusions:**

In women, higher testosterone levels increased the risk of iOAG. Both IOP-dependent and IOP-independent mechanisms may underlie this association. Managing testosterone levels may be particularly relevant for the prevention of neurodegeneration in the eye. Future research should confirm these findings.

**Supplementary Information:**

The online version contains supplementary material available at 10.1186/s13293-023-00512-z.

## Highlights

- Higher testosterone levels are associated with an increased incidence of open-angle glaucoma (OAG) in women, but not in men.
- In women, a genetic predisposition to develop higher levels of testosterone showed similar associations, internally validating our findings.
- In women, we observed an adverse association of testosterone levels with intraocular pressure (the main risk factor of OAG) as well as an inverse association with the retinal nerve fiber layer (thinned in OAG cases). Hereby, we confirmed the association between testosterone and OAG in women in an independent manner.
- These findings are important since the number of gender dysphoric individuals seeking hormonal therapy is increasing and OAG is a common sight-threatening disorder. If any, transgender men receiving testosterone therapy may have an increased risk of OAG.

**Supplementary Information:**

The online version contains supplementary material available at 10.1186/s13293-023-00512-z.

## Background

Glaucoma is a debilitating neurodegenerative eye disease that causes irreversible blindness. It currently affects more than 80 million people worldwide, among whom approximately 11 million are bilaterally blind [1]. The disease is characterized by thinning of the retinal nerve fiber layer (RNFL) and ganglion cell layer (GCL). Currently the only modifiable risk factor for glaucoma is the intraocular pressure (IOP) [2]. In the search for more modifiable risk factors, sex hormones have been named as possible targets [3, 4]. The two major sex hormones are testosterone and estrogen. A large part of the plasma testosterone is bound to either sex hormone-binding globulin (SHBG) or albumin, whereas only a small portion (0.5–3.0%) considered to be free testosterone. Both free testosterone and albumin-bound testosterone can be readily available to tissues, and are thus called bioavailable testosterone [5]. Not only are there differences in the circulating levels of the two sex hormones between men and women (e.g., circulating testosterone in men exceeds that of women by 15-fold) [6], they also have sex-specific biological implications. In men, testosterone deficiency is associated with obesity, metabolic syndrome, and type 2 diabetes, whereas women are predisposed to the same disorders when having an excess of testosterone [7].

Out of the two primary sex hormones, estrogen has been studied the most in the past (but only in women). Estrogen exposure has been inversely associated with IOP and open-angle glaucoma (OAG) risk [8, 9]. Similarly, women who went through an early menopause, an event significantly lowering estrogen levels, had a higher OAG risk [9–12]. This association was attributed to the increased ocular blood flow, decreased IOP, and neuroprotective effects associated with estrogen [3, 9]. While the effect of estrogen on IOP and glaucoma is relatively clear, the same does not apply to testosterone. Especially in men, only few studies looked at testosterone and IOP and those had conflicting results. Moreover, the mechanism(s) by which testosterone may influence the eyes are not yet understood. However, IOP-dependent and IOP-independent pathways, via ocular blood flow and neuroprotection, have been proposed as possible mechanisms by which testosterone may influence OAG risk [3].

While several studies investigated the association of testosterone and IOP, far less studies investigated the effect of testosterone on the risk of developing glaucoma. Therefore, we evaluated whether serum testosterone was associated with incidence of OAG, IOP, RNFL and GCL-inner plexiform layer (IPL). To assess causality, we also used the genetic risk for higher testosterone as proxy for measured testosterone in the association with OAG.

## Methods

### Study population

The participants in this study were collected from three independent cohorts from the Rotterdam Study (RS-I, RS-II, RS-III). This is a prospective population-based study including persons aged 45 years and over, who are living in the Ommoord district of Rotterdam, the Netherlands. Since 1991, there were 8679 participants who underwent ophthalmic and OAG examinations. Of these, 7898 participants had baseline data for sex hormone serum levels available. Genotyping was performed in 6873 of those participants. During follow-up, with visits taking place every 4–5 years [13], 187 of 7898 participants developed OAG.

### Ophthalmic assessment

Visual field testing was performed on all participants using the Humphrey Field Analyzer (HFA II 740; Carl Zeiss, Oberkochen, Germany). With Goldmann applanation tonometry, three IOP measurements were done on each eye, from which the median was used as the definite IOP. Optical coherence tomography (OCT; Topcon 3D OCT-2000, Topcon Optical Company, Tokyo Optical Co, Tokyo, Japan) imaging was performed in a small subset of the study population. Images were taken centered on optic disc (men: *N* = 255, women: *N* = 384) and the macula (men: *N* = 513, women: *N* = 709). The optic nerve head and macula were scanned in the horizontal direction in an area of 6 × 6 × 2.30 mm with 512 × 512 × 885 voxels, enabling us to detect structures with 5-μm resolution [14]. Retinal layer thicknesses were calculated automatically by Topcon’s built-in segmentation algorithm [15]. As the RNFL, GCL, and IPL are known to be affected in glaucoma [16], we used these retinal layer measurements. The RNFL thickness was measured from the internal limiting membrane to the inner boundary of the GCL. The thickness of the combined GCL and IPL was referred to as the GCL + layer [17]. Incident OAG (iOAG) was defined as glaucomatous visual field loss (GVFL) in at least one eye during follow-up with no prior known GVFL at baseline [18]. The definition of iOAG was independent of IOP and RNFL/GCL + . For iOAG cases, the affected eye was used in the analyses of IOP, RNFL, and GCL + . If both eyes were either affected or unaffected by GVFL, a random eye was chosen.

### Genotyping and imputation

DNA extraction was performed using whole blood samples following standardized and previously described protocols [19]. Genotyping was performed using both the Infinium II HumanHap550(-Duo) (RS-I & RS-II) and 610-Quad Genotyping BeadChip (RS-I & RS-III; Illumina, San Diego, CA, USA). Imputation of the RS datasets to the TOPMed reference panel was performed [20].

### Testosterone genetic risk score and trait

To calculate the sex-specific genetic risk scores (GRS), we used 855 SNPs associated with higher total and/or bioavailable testosterone in men and/or women as described by Ruth et al. [21]. About 80% of these SNPs (*N* = 696) were available in RS. The unavailability of the other SNPs was explained by insertions and deletions (54–63%), rare variants (minor allele frequency < 0.01; 5–9%), and less common variants (0.01 < minor allele frequency < 0.05; 0–4%). They were missing at random and evenly distributed over the chromosomes. In total, four different GRS were calculated: a GRS for higher total testosterone in men, a GRS for higher bioavailable testosterone in men, a GRS for higher total testosterone in women, and a GRS for higher bioavailable testosterone in women. A complete list of SNPs included in each GRS is available in Additional file 1. Serum level measurements of sex hormones were done on fasting blood samples, collected at the baseline examinations at the research center. All fasting blood samples used in our study were collected between 8.00 AM and 11.30 AM. Testosterone was measured with liquid chromatography–tandem mass spectrometry on a Waters XEVO TQ-S system (Waters, Milford, MA, USA) using the CHSMSMS Steroids Kit (Perkin Elmer, Turku, Finland) [22]. We excluded women with a total testosterone of > 10 nmol/L [23]. Bioavailable testosterone and free testosterone were calculated from total testosterone, SHBG, and albumin levels using the Vermeulen Equation [24]. SHBG was measured using the Immulite 2000XPi platform (Siemens, Los Angeles, CA, USA) [22]. Albumin concentration was set to 43 g/L for all participants, as is routine in clinical practice.

### Covariates

The participants’ height and weight were measured at the research center, and their body mass index (BMI) was calculated as weight in kilograms divided by height in squared meters. Validated food frequency questionnaires were filled out by the participants at baseline [20]. Energy intake in kcal/day was calculated based on these questionnaires. Participants with unreliable energy intake of < 500 or > 5000 kcal/day were excluded. Physical activity was assessed using a validated adapted version of either the Zutphen Physical Activity Questionnaire [25] or the LASA Physical Activity Questionnaire [26] and expressed in metabolic equivalent of task (MET) hours per week. Since the total activity scores from these questionnaires are not one on one comparable, standardized z-scores were used instead. Questionnaires were used to assess the smoking status of the participants. Participants were classified as never-smoker, former smoker, or current smoker. Hypertension was defined as a resting blood pressure exceeding 140/90 mmHg or the use of blood pressure-lowering medication. The use of diuretics, beta-blockers, calcium channel blockers, and renin–angiotensin–aldosterone system modifying agents were considered as blood pressure-lowering medication [27]. Diabetes mellitus was defined as either the fasting serum glucose exceeding 7.0 mmol/L, the non-fasting serum glucose level exceeding 11.1 mmol/L, or the use of blood glucose-lowering medication [28]. General practitioners’ records and hospital discharge letters were also monitored for presence of diabetes. Total cholesterol measurements were done on fasting serum samples with the Hitachi automatic analyzer (Boehringer Mannheim) [29].

### Statistical analysis

Independent t-tests and Chi-square tests were performed to analyze differences in the baseline characteristics. We performed multivariable logistic regression analyses to calculate odds ratios (ORs) with corresponding 95% confidence intervals (CIs) for the associations with iOAG. Multivariable linear regression was used to assess the associations with IOP, RNFL, and GCL + . Since testosterone levels are sex-specific [30], analyses were stratified on sex. The analyses were adjusted for age, BMI, and follow-up time. Follow-up time was determined as the time in years between baseline and the first visit with an iOAG diagnosis or until the last visit with reliable ophthalmic examination. Additional adjustment for lifestyle factors (physical activity, energy intake, and smoking status) or comorbidities (hypertension, diabetes mellitus and hypercholesterolemia) was also performed. Analyses were performed for total testosterone, bioavailable testosterone and free testosterone. The analyses for total testosterone were additionally adjusted for SHBG. Additionally, we modeled the GRS in quartiles with the first quartile (Q1) as reference category to test for evidence of linear trends. The median value for each category as continuous variables was used in separate logistic regression models. There are glaucoma factors that are affected by menopause [31, 32]. Therefore, we performed sensitivity analyses, evaluating the associations in postmenopausal women only (*N* = 4074). Age at menopause was assessed at baseline using a questionnaire, and was defined in retrospect as the age at final menstrual period, followed by a 12-month period of amenorrhea [33]. IBM SPSS statistics v28.0 for Windows (SPSS, Chicago, Illinois) was used for all statistical analyses. Statistical significance level was set at *p* < 0.05.

## Results

### Associations in men

The baseline characteristics of men who did and did not develop iOAG over time are depicted in Table 1. As expected men with iOAG were significantly older and had a higher IOP than men without iOAG. There was also a difference in RNFL and GCL + , although not statistically significant. Additionally, men who developed iOAG had significantly lower bioavailable and free testosterone.

**Table 1:** Baseline characteristics of men and women, stratified by incident open-angle glaucoma during follow-up

	Men			Women		
	No iOAG (*N* = 3321)	iOAG (*N* = 92)	*P*-value	No iOAG (*N* = 4390)	iOAG (*N* = 95)	*P*-value
Age, years	61.9 (7.3)	65.9 (6.8)	< 0.001*	62.5 (7.9)	65.6 (7.3)	< 0.001*
IOP, mmHg	14.1 (3.0)	16.3 (3.9)	< 0.001*	14.1 (2.9)	16.1 (3.8)	< 0.001*
Peripapillary RNFL, μm^a^	77.2 (17.2)	60.2 (16.9)	0.06	80.4 (16.1)	58.9 (36.9)	< 0.001*
Peripapillary GCL + , μm^a^	39.8 (9.3)	31.7 (14.8)	0.34	40.9 (10.1)	29.6 (22.1)	0.03*
Macular RNFL, μm^a^	32.7 (7.9)	27.0 (15.4)	0.32	32.7 (7.9)	19.8 (21.8)	0.10
Macular GCL + , μm^a^	69.5 (9.0)	61.5 (13.6)	0.19	69.6 (8.8)	54.8 (22.5)	0.007*
BMI, kg/m^2^	26.8 (3.4)	26.2 (2.8)	0.08	27.1 (4.4)	26.3 (3.7)	0.09
Follow-up time, years	9.7 (5.3)	10.2 (5.3)	0.40	9.9 (5.5)	10.8 (5.5)	0.14
Current smoker, N (%)	880 (26.6)	22 (23.9)	0.39	854 (19.5)	21 (22.1)	0.81
Physical activity, MET hours/week	− 0.2 (0.9)	− 0.2 (0.9)	0.98	0.2 (0.9)	0.3 (0.9)	0.20
Energy intake, kcal/day	2375.5 (608.5)	2325.7 (489.8)	0.50	1932.1 (521.0)	1796.7 (413.7)	0.02*
Hypertension, N (%)	1816 (55.0)	53 (58.9)	0.27	2297 (52.3)	48 (50.5)	0.43
Diabetes mellitus, N (%)	644 (19.5)	16 (17.4)	0.37	656 (14.9)	20 (21.1)	0.08
Cholesterol, mmol/L	5.9 (1.2)	6.0 (1.1)	0.37	6.3 (1.2)	6.5 (1.3)	0.10
Total testosterone, nmol/L	17.3 (6.0)	16.8 (6.5)	0.41	0.9 (0.6)	1.0 (0.8)	0.18
Bioavailable testosterone, nmol/L	6.9 (2.1)	6.1 (2.1)	< 0.001*	0.3 (0.2)	0.3 (0.3)	0.22
Free testosterone, ng/dL	8.5 (2.6)	7.5 (2.5)	< 0.001*	0.3 (0.2)	0.4 (0.3)	0.22
GRS total testosterone	10.0 (0.3)	10.1 (0.3)	0.02*	10.0 (0.3)	10.0 (0.2)	0.51
GRS bioavailable testosterone	5.3 (0.2)	5.3 (0.2)	0.68	6.5 (0.2)	6.6 (0.2)	0.04*

Characteristics are presented as mean (standard deviation) unless stated otherwise. *P-value < 0.05; ^a^Data only available for a small subset of participants and presented as median (interquartile range). *iOAG* incident open-angle glaucoma, *N* number, *IOP* intraocular pressure, *RNFL* retinal nerve fiber layer, *GCL +* ganglion cell layer + inner plexiform layer, *BMI* body mass index, *MET* metabolic equivalent of task, *GRS* genetic risk score

**Fig. 1:**
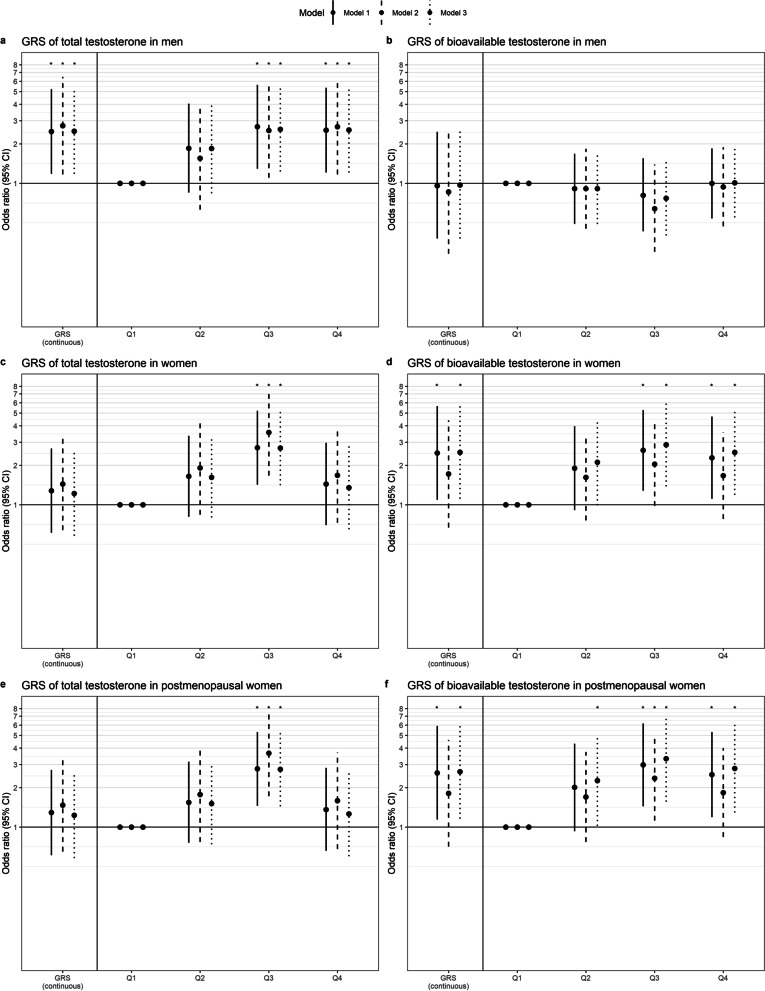
Odds ratios (95% confidence interval (CI)) for open-angle glaucoma stratified by sex, analyzed using logistic regression. Result for continuous genetic risk scores (GRS) for higher total testosterone (**a**, **c**, **e**) and higher bioavailable testosterone (**b**, **d**, **f**) are depicted on the left part of the figures. The right part of the figures depicts the results of the GRS for higher total testosterone (**a**, **c**, **e**) and higher bioavailable testosterone (**b**, **d**, **f**) per quartiles (Q). Model 1: adjusted for age, body mass index, and follow-up time. Model 2: model 1 additionally adjusted for physical activity, energy intake, and smoking status. Model 3: model 1 additionally adjusted for hypertension, diabetes mellitus, and total cholesterol. *P-value < 0.05

Total testosterone was not significantly associated with iOAG in men (Table 2, model 1, OR [95% CI]: 0.99 [0.95; 1.03] per nmol/L). Neither were bioavailable and free testosterone. An association between genetically higher levels of testosterone and iOAG would be more likely to represent causality. Therefore, we first tested the utility of the GRS for higher testosterone by looking at the association of the GRS and the different testosterone measurements, which were all highly significantly associated (P-value < 0.001). Contradicting the null-association between testosterone and iOAG, the GRS for higher total testosterone was significantly associated with a higher incidence of OAG (Fig. 1a, model 1; OR [95% CI]: 2.48 [1.18; 5.22] per unit). Especially men with a high GRS (quartile 3 and quartile 4) had a significantly increased risk of iOAG (OR [95% CI]: 2.70 [1.29; 5.62] and OR [95% CI]: 2.54 [1.21; 5.35], respectively) compared to men with a low GRS (quartile 1; P-trend = 0.009). We did not observe a significant association between the GRS for higher bioavailable testosterone and iOAG risk (Fig. 1b).

**Table 2:** Multivariable logistic and linear regression analyses for the association of testosterone measurements with glaucoma (-associated parameters) in men

		Total testosterone (per nmol/L)		Bioavailable testosterone (per nmol/L)		Free testosterone (per ng/dL)	
		OR (95% CI)	*P*	OR (95% CI)	*P*	OR (95% CI)	*P*
OAG	Model 1	0.99 (0.95; 1.03)	0.47	0.92 (0.83; 1.03)	0.16	0.94 (0.86; 1.03)	0.16
	Model 2	1.00 (0.96; 1.05)	0.93	0.97 (0.86; 1.10)	0.66	0.98 (0.88; 1.08)	0.66
	Model 3	0.98 (0.94; 1.02)	0.40	0.92 (0.82; 1.03)	0.13	0.93 (0.85; 1.02)	0.13

		Beta (95% CI)	*P*	Beta (95% CI)	*P*	Beta (95% CI)	*P*
IOP	Model 1	0.02 (− 0.01; 0.04)	0.22	0.03 (− 0.03; 0.09)	0.31	0.03 (− 0.02; 0.08)	0.31
	Model 2	0.03 (0.00; 0.05)	0.09	0.05 (− 0.02; 0.12)	0.17	0.04 (− 0.02; 0.10)	0.17
	Model 3	0.02 (− 0.01; 0.04)	0.16	0.04 (− 0.02; 0.10)	0.19	0.03 (− 0.02; 0.08)	0.19
Peripapillary RNFL	Model 1	0.91 (− 0.62; 2.44)	0.24	1.97 (− 1.30; 5.25)	0.24	1.60 (− 1.06; 4.27)	0.24
	Model 2	1.19 (− 0.48; 2.86)	0.16	2.62 (− 0.97; 6.21)	0.15	2.13 (− 0.79; 5.05)	0.15
	Model 3	0.94 (− 0.60; 2.48)	0.23	1.94 (− 1.36; 5.23)	0.25	1.58 (− 1.11; 4.26)	0.25
Peripapillary GCL +	Model 1	0.60 (− 0.25; 1.44)	0.17	1.41 (− 0.40; 3.22)	0.13	1.15 (− 0.32; 2.62)	0.13
	Model 2	0.77 (− 0.15; 1.69)	0.10	1.86 (− 0.11; 3.82)	0.06	1.51 (− 0.09; 3.11)	0.06
	Model 3	0.60 (− 0.25; 1.45)	0.17	1.38 (− 0.44; 3.21)	0.14	1.13 (− 0.36; 2.61)	0.14
Macular RNFL	Model 1	1.07 (− 0.31; 2.45)	0.13	2.23 (− 0.68; 5.14)	0.13	1.82 (− 0.55; 4.18)	0.13
	Model 2	1.83 (0.34; 3.32)	0.02*	3.53 (0.43; 6.63)	0.03*	2.87 (0.35; 5.39)	0.03*
	Model 3	1.09 (− 0.30; 2.49)	0.13	2.23 (− 0.71; 5.17)	0.14	1.81 (− 0.58; 4.20)	0.14
Macular GCL +	Model 1	0.46 (− 0.33; 1.25)	0.25	1.00 (− 0.66; 2.66)	0.24	0.81 (− 0.54; 2.17)	0.24
	Model 2	0.88 (0.15; 1.62)	0.02*	1.71 (0.18; 3.24)	0.03*	1.39 (0.15; 2.63)	0.03*
	Model 3	0.46 (− 0.34; 1.26)	0.26	0.97 (− 0.71; 2.65)	0.26	0.79 (− 0.58; 2.15)	0.26

Model 1: adjusted for age, body mass index, and follow-up time. Model 2: model 1 additionally adjusted for physical activity, energy intake, and smoking status. Model 3: model 1 additionally adjusted for hypertension, diabetes mellitus, and total cholesterol. The analyses for total testosterone were additionally adjusted for sex hormone-binding globulin. * P-value < 0.05. Abbreviations: *OR* odds ratio, *CI* confidence interval, *OAG* open-angle glaucoma, *IOP* intraocular pressure, *RNFL* retinal nerve fiber layer, *GCL* *+*  ganglion cell layer + inner plexiform layer

We did not find a significant association between IOP at follow-up and total testosterone (Table 2, model 1, Beta [95% CI]: 0.02 [− 0.01; 0.04] per nmol/L). Similarly, bioavailable and free testosterone were not associated with IOP at follow-up.

A small number of men underwent OCT imaging (optic disc: *N* = 255, macula: *N* = 513). There was a tendency of larger peripapillary and macular RNFL and GCL + with higher total testosterone (Table 2). After additional adjustment for lifestyle factors (model 2), significant associations were observed between higher testosterone levels and larger macular RNFL thickness (Beta [95% CI]: 1.83 [0.34; 3.32] per nmol/L). Similar associations were present for the macular GCL + thickness ( Beta [95% CI]: 0.88 [0.15; 1.62] per nmol/L). Bioavailable testosterone and free testosterone showed comparable associations with RNFL and GCL +.

Additional adjustment of all aforementioned analyses for lifestyle factors (model 2) or for comorbidities (model 3) did not change the results, unless stated otherwise.

### Associations in women

The baseline characteristics of women who did and did not develop iOAG during follow-up are displayed in Table 1. Women with iOAG were significantly older and had a lower energy intake. As expected, women who developed iOAG had a higher IOP and thinner RNFL and GCL +. The baseline characteristics of only postmenopausal women (Table 3) were comparable to those of the total female population.

**Table 3:** Baseline characteristics of postmenopausal women, stratified by incident open-angle glaucoma during follow-up

	Postmenopausal women		
	No iOAG (N = 3981)	iOAG (N = 93)	*P*-value
Age, years	63.6 (7.4)	65.9 (7.1)	0.003*
IOP, mmHg	14.1 (2.9)	16.1 (3.9)	< 0.001*
Peripapillary RNFL, μm^a^	80.4 (15.6)	58.9 (36.9)	< 0.001*
Peripapillary GCL + , μm^a^	40.9 (9.5)	29.6 (22.1)	0.03*
Macular RNFL, μm^a^	32.2 (7.8)	19.8 (22.4)	0.13
Macular GCL + , μm^a^	69.6 (9.2)	54.8 (22.5)	0.009*
BMI, kg/m^2^	27.1 (4.3)	26.3 (3.7)	0.08
Follow-up time, years	10.3 (5.5)	10.8 (5.5)	0.40
Current smoker, N (%)	765 (19.2)	21 (22.6)	0.73
Physical activity, MET hours/week	0.2 (0.9)	0.3 (0.9)	0.23
Energy intake, kcal/day	1903.5 (502.3)	1791.1 (413.2)	0.05*
Hypertension, N (%)	2152 (54.1)	46 (49.5)	0.42
Diabetes mellitus, N (%)	630 (15.8)	20 (21.5)	0.15
Cholesterol, mmol/L	6.4 (1.2)	6.5 (1.3)	0.31
Total testosterone, nmol/L	0.9 (0.6)	1.1 (0.8)	0.17
Bioavailable testosterone, nmol/L	0.3 (0.2)	0.3 (0.3)	0.25
Free testosterone, ng/dL	0.3 (0.2)	0.4 (0.3)	0.25
GRS total testosterone	10.0 (0.3)	10.0 (0.3)	0.50
GRS bioavailable testosterone	6.5 (0.2)	6.6 (0.2)	0.03*

Characteristics are presented as mean (standard deviation) unless stated otherwise. *P-value < 0.05; ^a^ Data only available for a small subset of participants and presented as median (interquartile range). *iOAG* incident open-angle glaucoma, *N* number, *IOP* intraocular pressure, *RNFL* retinal nerve fiber layer, *GCL +* ganglion cell layer + inner plexiform layer; *BMI* body mass index, *MET* metabolic equivalent of task, *GRS* genetic risk score

After adjustment for multiple confounders, higher total testosterone tended to increase the risk of iOAG in women (Table 4, model 1, OR [95% CI]: 1.25 [0.97; 1.62] per nmol/L). Bioavailable testosterone (Table 4, model 1, OR [95% CI]: 2.05 [1.00; 4.18] per nmol/L) and free testosterone (Table 4, model 1, OR [95% CI]: 1.79 [1.00; 3.20] per ng/dL) were both significantly associated with a higher iOAG risk. Adjusting the aforementioned analyses for lifestyle factors strengthened the associations, causing the association between total testosterone and iOAG risk to reach statistical significance as well (Table 4, model 2, OR [95% CI]: 1.34 [1.05; 1.71] per nmol/L).

**Table 4:** Multivariable logistic and linear regression analyses for the association of testosterone measurements with glaucoma (-associated parameters) in women

		Total testosterone (per nmol/L)		Bioavailable testosterone (per nmol/L)		Free testosterone (per ng/dL)	
		OR (95% CI)	*P*	OR (95% CI)	*P*	OR (95% CI)	*P*
OAG	Model 1	1.25 (0.97; 1.62)	0.09	2.05 (1.00; 4.18)	0.05*	1.79 (1.00; 3.20)	0.05*
	Model 2	1.34 (1.05; 1.71)	0.02*	2.27 (1.12; 4.57)	0.02*	1.95 (1.10; 3.44)	0.02*
	Model 3	1.26 (0.97; 1.64)	0.09	2.04 (0.99; 4.20)	0.05	1.78 (0.99; 3.21)	0.05

		Beta (95% CI)	*P*	Beta (95% CI)	*P*	Beta (95% CI)	*P*
IOP	Model 1	0.11 (− 0.06; 0.29)	0.20	0.58 (0.05; 1.10)	0.03*	0.47 (0.04; 0.90)	0.03*
	Model 2	0.11 (− 0.08; 0.31)	0.26	0.59 (0.01; 1.17)	0.05*	0.48 (0.01; 0.96)	0.05*
	Model 3	0.09 (− 0.08; 0.27)	0.29	0.47 (− 0.07; 1.00)	0.09	0.38 (− 0.05; 0.82)	0.09
Peripapillary RNFL	Model 1	− 3.54 (− 7.02; − 0.06)	0.05*	− 5.70 (− 17.63; 6.24)	0.35	− 4.63 (− 14.34; 5.07)	0.35
	Model 2	− 3.37 (− 7.48; 0.74)	0.11	− 6.47 (− 20.28; 7.34)	0.36	− 5.26 (− 16.49; 5.97)	0.36
	Model 3	− 3.36 (− 6.89; 0.18)	0.06	− 5.15 (− 17.47; 7.16)	0.41	− 4.19 (− 14.21; 5.82)	0.41
Peripapillary GCL +	Model 1	− 2.18 (− 4.11; − 0.25)	0.03*	− 4.57 (− 11.21; 2.07)	0.18	− 3.72 (− 9.12; 1.68)	0.18
	Model 2	− 1.68 (− 3.92; 0.57)	0.14	− 3.83 (− 11.37; 3.72)	0.32	− 3.11 (− 9.25; 3.02)	0.32
	Model 3	− 2.01 (− 3.96; − 0.05)	0.05*	− 4.12 (− 10.96; 2.72)	0.24	− 3.35 (− 8.91; 2.22)	0.24
Macular RNFL	Model 1	− 3.30 (− 7.80; 1.21)	0.15	− 10.60 (− 25.59; 4.39)	0.17	− 8.62 (− 20.81; 3.57)	0.17
	Model 2	− 3.82 (− 9.11; 1.47)	0.16	− 12.99 (− 30.35; 4.37)	0.14	− 10.57 (− 24.68; 3.55)	0.14
	Model 3	− 3.41 (− 7.96; 1.14)	0.14	− 12.19 (− 27.52; 3.15)	0.12	− 9.91 (− 22.38; 2.56)	0.12
Macular GCL +	Model 1	− 1.85 (− 4.51; 0.81)	0.17	− 5.49 (− 14.34; 3.37)	0.23	− 4.46 (− 11.66; 2.74)	0.23
	Model 2	− 2.25 (− 5.32; 0.82)	0.15	− 7.46 (− 17.55; 2.62)	0.15	− 6.07 (− 14.27; 2.13)	0.15
	Model 3	− 1.79 (− 4.48; 0.90)	0.19	− 5.71 (− 14.77; 3.35)	0.22	− 4.65 (− 12.01; 2.72)	0.22

Model 1: adjusted for age, body mass index, and follow-up time. Model 2: model 1 additionally adjusted for physical activity, energy intake, and smoking status. Model 3: model 1 additionally adjusted for hypertension, diabetes mellitus, and total cholesterol. The analyses for total testosterone were additionally adjusted for sex hormone-binding globulin. *P-value < 0.05. *OR* odds ratio, *CI* confidence interval, *OAG* open-angle glaucoma, *IOP* intraocular pressure, *RNFL* retinal nerve fiber layer, *GCL +* ganglion cell layer + inner plexiform layer

The GRS for higher total and bioavailable testosterone were highly significantly associated with the testosterone measurements (*P*-value < 0.001). Secondly, we analyzed the association between the GRS for higher total and bioavailable testosterone and iOAG risk. There was a tendency of increased risk of iOAG with increased GRS for higher total testosterone (Fig. 1c), although not statistically significant. The GRS for higher bioavailable testosterone however was significantly associated with increased incidence of OAG (Fig. 1d, model 1; OR [95% CI]: 2.48 [1.09; 5.65] per unit). Especially women with a high GRS (quartile 3 and quartile 4) had an increased risk of iOAG (OR [95% CI]: 2.60 [1.28; 5.26] and OR [95% CI]: 2.28 [1.11; 4.69], respectively) compared to women with a low GRS (quartile 1) (P-trend = 0.02).

For IOP at follow-up as outcome (Table 4, model 1), we observed no significant associations with total testosterone (Beta [95% CI]: 0.11 [-0.06; 0.29] per nmol/L). However both bioavailable testosterone (Beta [95% CI]: 0.58 [0.05; 1.10] per nmol/L) and free testosterone (Beta [95% CI]: 0.47 [0.04; 0.90] per ng/dL) were significantly associated with increased IOP at follow-up.

In a subset of women (optic disc: *N* = 384, macula: *N* = 709), we assessed the association of testosterone with RNFL and GCL + . Higher total testosterone was associated with a decreased thickness of the peripapillary RNFL and GCL + (Table 4, model 1, Beta [95% CI]: − 3.54 [− 7.02; − 0.06] per nmol/L and Beta [95% CI]: − 2.18 [− 4.11; − 0.25] per nmol/L, respectively), but not with that of the macula (Beta [95% CI]: − 3.30 [− 7.80; 1.21] per nmol/L and Beta [95% CI]: − 1.85 [− 4.51; 0.81] per nmol/L, respectively). Neither bioavailable nor free testosterone were significantly associated with the peripapillary or macular RNFL and GCL + thickness, but did show the same tendency.

Adjusting the aforementioned analyses for lifestyle factors (model 2) or comorbidities (model 3) did not change the results, unless stated otherwise. Including only postmenopausal women in the analyses provided similar results (Table 5, Fig. 1e, f).

**Table 5:** Multivariable logistic and linear regression analyses for the association of testosterone measurements with glaucoma (-associated parameters) in postmenopausal women

		Total testosterone (per nmol/L)		Bioavailable testosterone (per nmol/L)		Free testosterone (per ng/dL)	
		OR (95% CI)	*P*	OR (95% CI)	*P*	OR (95% CI)	*P*
OAG	Model 1	1.26 (0.98; 1.63)	0.08	2.02 (0.99; 4.14)	0.05	1.77 (0.99; 3.17)	0.05
	Model 2	1.34 (1.05; 1.71)	0.02*	2.25 (1.12; 4.53)	0.02*	1.94 (1.10; 3.42)	0.02*
	Model 3	1.27 (0.98; 1.64)	0.07	2.03 (0.99; 4.17)	0.06	1.78 (0.99; 3.19)	0.06

		Beta (95% CI)	*P*	Beta (95% CI)	*P*	Beta (95% CI)	*P*
IOP	Model 1	0.13 (− 0.05; 0.31)	0.15	0.59 (0.04; 1.13)	0.04*	0.48 (0.03; 0.92)	0.04*
	Model 2	0.13 (− 0.07; 0.32)	0.22	0.57 (− 0.02; 1.17)	0.06	0.47 (− 0.02; 0.95)	0.06
	Model 3	0.11 (− 0.07; 0.29)	0.25	0.47 (− 0.08; 1.02)	0.09	0.38 (− 0.06; 0.83)	0.09
Peripapillary RNFL	Model 1	− 3.27 (− 6.78; 0.24)	0.07	− 4.52 (− 16.63; 7.59)	0.46	− 3.67 (− 13.52; 6.17)	0.46
	Model 2	− 2.99 (− 7.14; 1.16)	0.16	− 5.03 (− 19.04; 8.99)	0.48	− 4.09 (− 15.48; 7.31)	0.48
	Model 3	− 3.08 (− 6.65; 0.48)	0.09	− 4.01 (− 16.49; 8.46)	0.53	− 3.26 (− 13.41; 6.88)	0.53
Peripapillary GCL +	Model 1	− 1.96 (− 3.86; − 0.06)	0.04*	− 4.05 (− 10.60; 2.50)	0.23	− 3.29 (− 8.62; 2.04)	0.23
	Model 2	− 1.34 (− 3.55; 0.87)	0.23	− 3.09 (− 10.55; 4.37)	0.42	− 2.51 (− 8.58; 3.55)	0.42
	Model 3	− 1.85 (− 3.77; 0.08)	0.06	− 3.80 (− 10.52; 2.93)	0.27	− 3.09 (− 8.56; 2.39)	0.27
Macular RNFL	Model 1	− 3.41 (− 8.58; 1.77)	0.20	− 10.68 (− 27.91; 6.55)	0.22	− 8.68 (− 22.70; 5.33)	0.22
	Model 2	− 4.01 (− 10.10; 2.08)	0.20	− 13.48 (− 33.42; 6.47)	0.19	− 10.96 (− 27.18; 5.26)	0.19
	Model 3	− 3.62 (− 8.87; 1.62)	0.18	− 12.55 (− 30.18; 5.08)	0.16	− 10.21 (− 24.55; 4.13)	0.16
Macular GCL +	Model 1	− 1.74 (− 4.74; 1.26)	0.26	− 4.87 (− 14.87; 5.13)	0.34	− 3.96 (− 12.09; 4.17)	0.34
	Model 2	− 2.10 (− 5.58; 1.37)	0.24	− 6.87 (− 18.26; 4.52)	0.24	− 5.59 (− 14.85; 3.67)	0.24
	Model 3	− 1.79 (− 4.83; 1.25)	0.25	− 5.53 (− 15.75; 4.70)	0.29	− 4.49 (− 12.81; 3.82)	0.29

Model 1: adjusted for age, body mass index, and follow-up time. Model 2: model 1 additionally adjusted for physical activity, energy intake, and smoking status. Model 3: model 1 additionally adjusted for hypertension, diabetes mellitus, and total cholesterol. The analyses for total testosterone were additionally adjusted for sex hormone-binding globulin. *P-value < 0.05. *OR* odds ratio, *CI* confidence interval, *OAG* open-angle glaucoma,; *IOP* intraocular pressure, *RNFL* retinal nerve fiber layer, *GCL +* ganglion cell layer + inner plexiform layer

## Discussion

In this prospective population-based study we found that a higher GRS for higher total testosterone, but not total testosterone itself, was associated with an increased risk of iOAG in men. In women, we found significant adverse associations between testosterone levels and iOAG risk. Similarly, a higher GRS for higher bioavailable testosterone was also associated with increased iOAG risk. Moreover, we observed an adverse association between testosterone and IOP, along with an inverse association between testosterone and RNFL and GCL +. The association between testosterone and iOAG in women may therefore be explained by both IOP-dependent and IOP-independent mechanisms.

While testosterone is primarily known as a male sex hormone and estrogen as a female sex hormone, both are present in every human body [30, 34]. Although the normal value serum levels differ between sexes, both hormones are essential for a healthy functioning body. They affect multiple organs such as the brain and the heart, and influence fertility, muscle mass, fat distribution, red blood cell production, and blood sugar regulation [34–36]. Testosterone can be found as three different forms in the circulation: the inactive SHBG-bound testosterone, the mildly active albumin-bounded testosterone, and the active free testosterone [37]. Free testosterone and albumin-bounded testosterone is collectively called the bioavailable testosterone. This is the fraction of the total testosterone that can move to tissues and exert its effect [38]. The testosterone that is bound to SHBG is biologically inactive [39] and thus the concentration of total testosterone might not always reflect the true androgen status [40]. In multiple epidemiologic studies, more robust associations with androgen-dependent outcomes were found for bioavailable and free testosterone than for total testosterone [41]. In the present study, we therefore not only looked at associations with total testosterone, but also both bioavailable testosterone and free testosterone. By doing so, we showed that all three forms of testosterone were associated with higher iOAG risk in women. We also confirmed the association by using two GRS for higher testosterone as proxies of actual testosterone levels and found similar associations. To the best of our knowledge, we are the first to show a significant adverse association between GRS for higher testosterone levels and iOAG risk in women. Previous research assessing the association between testosterone and the risk of developing glaucoma is scarce. To date, only the Nurses’ Health Study investigated the association between testosterone and glaucoma [42]. This study observed that in postmenopausal women, higher total testosterone was associated with a significantly increased OAG risk (T2 vs. T1; OR [95%]: 2.03 [1.16; 3.56] and T3 vs. T1; OR [95% CI]: 1.84 [1.02; 3.33]), which is consistent with the results of our study.

The direct association between testosterone levels and glaucoma has not been investigated yet in men. However, one recent study investigated the effects of androgen-deprivation therapies (ADT) on primary OAG [43]. They found that ADT in men with prostate cancer reduced the risk of primary OAG, suggesting that reducing testosterone levels may protect against primary OAG. In our study, we did not find a significant association between testosterone and OAG in men. This may be explained by the fact that ADT, a gonadotropin-releasing hormone agonist or antagonist, influences not only testosterone levels, but also that of other androgens [44]. The protective effect of ADT might therefore be explained by the levels of other androgens, rather than that of testosterone.

Contradicting findings have been reported for the association between IOP and testosterone in men. Intramuscular testosterone injections had no effect on the IOP in men with idiopathic hypogonadotropic hypogonadism [45], which is consistent with our results. However, in obese and overweight men IOP was found to be significantly correlated with testosterone levels [46]. In the present study, we did not find associations with IOP when analyzing men with a BMI above 25 (data not shown, *P*-value > 0.19). The results for women in past literature are more consistent. Three studies have provided evidence that testosterone levels are adversely associated with IOP [42, 47, 48]. These studies only included postmenopausal women. In our study, only participants above the age of 45 were included, with a mean age in women with iOAG of 65.6 years, and mean age in women without iOAG of 62.5 years. As the mean age of menopause is 50.5 years, it can be assumed that most of the participants in our study were also postmenopausal, which would make our results relatively comparable. Furthermore, testosterone levels are not associated with menopause [49–51]. IOP however is significantly higher after than before menopause with the difference in IOP being around 3 mmHg [31]. In the present study, it was indeed found that premenopausal women had a significantly lower IOP at baseline (mean ± SD: 13.4 ± 2.8 mmHg) than postmenopausal women (mean ± SD: 14.2 ± 3.0 mmHg; *P*-value < 0.001), although this difference was smaller than described in the literature. In both the primary and sensitivity analyses, we reported an adverse association of bioavailable and free testosterone with IOP, confirming earlier reported findings.

The role of testosterone in the pathogenesis of OAG has yet to be determined. IOP-dependent and IOP-independent pathways, via ocular blood flow and neuroprotection, have been described as possible mechanisms by which testosterone may influence iOAG risk [3]. However, results regarding the vascular effects of testosterone have been contradictory. Toker et al. found that in premenopausal women higher testosterone levels were associated with an increased resistive index, reduced peak systolic flow, and reduced end systemic flow in the central retinal artery [47]. The negative influence testosterone had on ocular blood flow was further increased in postmenopausal women. More recently, Alpogan et al. did not observe a difference in ocular blood flow in the central retinal artery or ocular artery in transgender men receiving testosterone supplements [52]. The neuroprotective effect of testosterone has been scarcely investigated, but unlike the ocular blood flow, was more consistent with testosterone being associated with an increased RNFL thickness and neuroprotection [52, 53]. In the present study, we confirmed these findings, but only in men, where higher testosterone tended to be associated with larger RNFL and GCL +. However, in women we observed a significant association between higher testosterone and thinner peripapillary RNFL and GCL +. These findings, in combination with the adverse association between testosterone and OAG risk (in women only), suggest another mechanism may override its possible neuroprotective effect.

Our findings are in line with results on other diseases and symptoms, where high testosterone seems to affect men and women differently [54, 55]. For example, high testosterone levels are harmful in women but not in men, while low testosterone levels are harmful in men but not in women. It is important to note that testosterone also influences the presence of other comorbidities in a sex-dependent manner, and these comorbidities may subsequently affect the risk of OAG. However, despite the apparent associations of testosterone with hypertension [56–58], diabetes mellitus [59–63], and cholesterol levels [64–66], and the known or suggested associations of these comorbidities with glaucoma, additional adjustment for these comorbidities did not clearly change the observed associations. Future research should aim to unravel the role of testosterone in the pathogenesis of OAG, keeping these sex-differences in mind.

This study has several strengths. It used a prospective population-based study design with a long follow-up, with visits taking place every 4–5 years. Only visual field testing, and not IOP, RNFL and GCL +, was included in the definition of iOAG. Therefore, by analyzing the RNFL and GCL + of the participants, regardless of iOAG diagnosis, we were able to confirm the association between testosterone and iOAG in an independent manner. We used two GRS as proxies for the actual measured testosterone levels, allowing us to internally validate our findings. Additionally, testosterone measurements were done with liquid chromatography–tandem mass spectrometry which has been proven to measure more precise testosterone levels than immunoassay, especially in women [67]. We also adjusted for a broad range of covariates to reduce confounding bias. Previous studies have suggested that increased physical activity is associated with increased serum testosterone levels [68–70]. Moreover, obesity and low serum testosterone levels are interrelated and both strongly associated by dietary factors [71, 72]. Lastly, active smokers have significantly higher testosterone levels than non-smokers [73–75]. As these lifestyle factors have also been suggested to be associated with the risk of glaucoma, we considered these as important possible confounding factors. Indeed, additional adjustment with lifestyle factors (physical activity, energy intake, and smoking status) strengthened some of the observed associations. This effect was mainly caused by the addition of energy intake to the model. Adding physical activity or smoking status to the model did not clearly change the associations. Since OAG (higher), energy intake (lower), and testosterone levels (lower) are all associated with higher age, it is possible that effect modification is present. When stratifying our analyses on energy intake (data not shown), the association of testosterone with macular RNFL was especially present in men with a lower than average energy intake. Similarly, the association of bioavailable testosterone with iOAG was stronger in women with a lower than average energy intake. When stratifying our analyses on age (data not shown), the association of testosterone with macular RNFL was especially present in men with a higher than average age. Also, the association of bioavailable testosterone with iOAG was stronger in women with a higher than average age. Thus, for both men and women, stratifying on energy intake or age provided results in the same direction. These considerations should be kept in mind when interpreting the results of our study.

Limitations included the relatively low number of iOAG cases. A population-based study gives a good representation of the population, but consequently few iOAG cases, as OAG has a low incidence in the general population [18]. Having a female population of both premenopausal women and postmenopausal women could also be considered a limitation as menopause affects IOP and other glaucoma factors [31, 32]. However, we performed sensitivity analyses in postmenopausal women only to cope with this limitation. Lastly, as testosterone and estrogen are correlated [76], we cannot definitively state that the associations observed in our study are purely caused by testosterone. The lack of reliable estrogen data is therefore a noteworthy limitation.

## Perspectives and significance

The sex hormones have been posed as potential modifiable risk factors for OAG. Previous research has primarily focused on estrogen, which has been inversely associated with OAG and IOP. The association between testosterone and the risk of OAG has not been studied in detail. We observed sex-specific associations between testosterone and OAG. Given that the number of gender dysphoric individuals seeking hormonal therapy is increasing, and OAG is a common sight-threatening disorder, we believe that it is important to share these intriguing findings with a broad public. If any, transgender men receiving testosterone therapy may have an increased risk of OAG.

## Conclusions

The results of this study suggest an adverse association between testosterone and iOAG risk in women. A high risk for iOAG was especially seen for women with a high GRS for higher bioavailable testosterone. Both IOP-dependent and IOP-independent may underlie these associations. These findings are potentially important given that transgender men may already have a genetic predisposition for an increased risk of iOAG, and receiving testosterone therapy may exacerbate this risk even further. If further studies confirm this association, this may impact hormone therapy for transgender persons.

## Supplementary Information

**Additional file 1**. Single nucleotide polymorphisms included in the sex-specific genetic risk scores of total testosterone and bioavailable testosterone.

## Data Availability

Data can be obtained upon request. Requests should be directed towards the management team of the Rotterdam Study (datamanagement.ergo@erasmusmc.nl), which has a protocol for approving data requests. Due to restrictions based on privacy regulations and informed consent of the participants, data cannot be made freely available in a public repository.
