# Supplementary material for: Higher testosterone is associated with open-angle glaucoma in women: a genetic predisposition?
Source: Biol Sex Differ. 2023 May 9;14:27. doi: 10.1186/s13293-023-00512-z (PMC10170716; doi:10.1186/s13293-023-00512-z)
Supplement: Supplementary file 1 — Additional file 1. Single nucleotide polymorphisms included in the sex-specific genetic risk scores of total testosterone and bioavailable testosterone. [file 13293_2023_512_MOESM1_ESM.pdf]

Additional file 1. Single nucleotide polymorphisms (SNPs) included in the sex-specific genetic risk scores of total testosterone and bioavailable testosterone.

SNP	Effect Allele for GRS of total testosterone in men	Beta for GRS of total testosterone in men	Effect Allele for GRS of bioavailable testosterone in men	Beta for GRS of bioavailable testosterone in men	Effect Allele for GRS of total testosterone in women	Beta for GRS of total testosterone in women	Effect Allele for GRS of bioavailable testosterone in women	Beta for GRS of bioavailable testosterone in women
rs618888	T	0.0231623	T	0.0284465	T	0.00774245	T	0.0155739
rs10892924	T	0.0316012	T	0.0393968	T	0.0107634	T	0.0187203
rs2011425	T	0.0249772	T	0.0502524	T	0.0334892	T	0.0376561
rs1812755	T	0.0257744	T	0.033863	-	-	T	0.0136023
rs10192634	T	0.0231899	T	0.0166649	-	-	T	0.0166649
rs66956368	T	0.0304555	T	0.0335677	-	-	T	0.0167712
rs7915430	T	0.0254373	T	0.020841	-	-	-	-
rs34040779	T	0.0335641	T	0.0348252	-	-	-	-
rs11703376	T	0.0342623	T	0.0396526	-	-	-	-
rs28929474	T	0.221657	C	0.0915661	T	0.0399493	C	0.0915661
rs5855544	T	0.0130571	-	-	T	0.0333264	T	0.0151304
rs1171617	T	0.0171923	-	-	T	0.0522618	T	0.029502
rs113247979	T	0.0471068	-	-	T	0.139703	T	0.0822416
rs61755050	T	0.21076	-	-	T	0.0617191	C	0.0909082
rs6073431	T	0.0309435	-	-	-	-	C	0.0145654
rs4826631	T	0.00635168	-	-	T	0.00635168	-	-
rs2675611	T	0.00718597	-	-	T	0.00718597	-	-
rs4835948	T	0.0074577	-	-	T	0.0074577	-	-
rs2256191	T	0.00750751	-	-	T	0.00750751	-	-
rs1782652	T	0.00761397	-	-	T	0.00761397	-	-
rs733190	T	0.00773479	-	-	T	0.00773479	-	-
rs1553668	T	0.00780902	-	-	T	0.00780902	-	-
rs11629457	T	0.00808494	-	-	T	0.00808494	-	-
rs881301	T	0.0081257	-	-	T	0.0081257	-	-
rs11556924	T	0.00823325	-	-	T	0.00823325	-	-
rs768863	T	0.00833812	-	-	T	0.00833812	-	-

rs941446	T	0.00843126	-	-	T	0.00843126	-	-
rs10982192	T	0.0238068	-	-	T	0.00855494	-	-
rs11023881	T	0.00857457	-	-	T	0.00857457	-	-
rs11564722	T	0.00868151	-	-	T	0.00868151	-	-
rs139763690	T	0.00928587	-	-	T	0.00928587	-	-
rs12294104	T	0.00933327	-	-	T	0.00933327	-	-
rs1128249	T	0.00940047	-	-	T	0.00940047	-	-
rs782207398	T	0.00946207	-	-	T	0.00946207	-	-
rs10278686	T	0.00987923	-	-	T	0.00987923	-	-
rs7759938	T	0.00999899	-	-	T	0.00999899	-	-
rs77017252	T	0.0104464	-	-	T	0.0104464	-	-
rs6130613	T	0.0105361	-	-	T	0.0105361	-	-
rs2241388	T	0.0108006	-	-	T	0.0108006	-	-
rs61823391	T	0.0110331	-	-	T	0.0110331	-	-
rs11102518	T	0.0114067	-	-	T	0.0114067	-	-
rs111861797	T	0.0115251	-	-	T	0.0115251	-	-
rs62394296	T	0.01233	-	-	T	0.01233	-	-
rs1977658	T	0.0145824	-	-	T	0.0145824	-	-
rs45448191	T	0.0180022	-	-	T	0.0180022	-	-
rs2306847	T	0.0189427	-	-	T	0.0189427	-	-
rs8126001	T	0.00953407	-	-	T	0.0196947	-	-
rs41284816	T	0.0260423	-	-	T	0.0260423	-	-
rs2517582	T	0.0135974	-	-	T	0.0277124	-	-
rs10740131	T	0.0380455	-	-	T	0.0283387	-	-
rs2061679	T	0.0332963	-	-	T	0.0332963	-	-
rs4961485	T	0.0157393	-	-	T	0.0391398	-	-
rs146497684	T	0.0442434	-	-	T	0.0442434	-	-
rs77822621	T	0.0174868	-	-	T	0.0479737	-	-
rs4431325	T	0.0206225	-	-	T	0.0496715	-	-
rs577721086	T	0.0196674	-	-	T	0.0554554	-	-
rs72660136	T	0.0212318	-	-	T	0.0604855	-	-
rs9461224	T	0.0180325	-	-	-	-	-	-
rs1708302	T	0.0182046	-	-	-	-	-	-

rs36086195	T	0.0188837	-	-	-	-	-	-
rs1229498	T	0.0189866	-	-	-	-	-	-
rs2721195	T	0.020374	-	-	-	-	-	-
rs10421262	T	0.02094	-	-	-	-	-	-
rs11099675	T	0.021099	-	-	-	-	-	-
rs7610366	T	0.0211166	-	-	-	-	-	-
rs61922185	T	0.0214027	-	-	-	-	-	-
rs1421085	T	0.0215477	-	-	-	-	-	-
rs6870458	T	0.0229601	-	-	-	-	-	-
rs1033667	T	0.0234566	-	-	-	-	-	-
rs631695	T	0.0242925	-	-	-	-	-	-
rs1058319	T	0.024331	-	-	-	-	-	-
rs13074711	T	0.0280018	-	-	-	-	-	-
rs2905801	T	0.02876	-	-	-	-	-	-
rs12614829	T	0.0302107	-	-	-	-	-	-
rs540730	T	0.030322	-	-	-	-	-	-
rs6766859	T	0.0305299	-	-	-	-	-	-
rs12406721	T	0.0332823	-	-	-	-	-	-
rs10868080	T	0.0358052	-	-	-	-	-	-
rs11735092	T	0.0371495	-	-	-	-	-	-
rs1933801	T	0.0375388	-	-	-	-	-	-
rs41310053	T	0.111733	-	-	-	-	-	-
rs114816312	T	0.119397	-	-	-	-	-	-
rs145602600	T	0.132605	-	-	-	-	-	-
rs7314285	G	0.0389623	T	0.0254155	-	-	T	0.0375233
rs12658172	G	0.0177375	G	0.018886	G	0.0516979	G	0.0303213
rs590097	G	0.0192827	G	0.018975	G	0.0606189	G	0.0339639
rs12810788	G	0.0247636	G	0.0270491	-	-	G	0.0152622
rs7679843	G	0.0433159	G	0.0501614	-	-	G	0.0235565
rs1994721	G	0.0492752	G	0.0335511	-	-	G	0.0335511
rs4678408	G	0.00937888	G	0.0261471	G	0.00937888	-	-
rs61762319	G	0.0607202	G	0.0499764	-	-	-	-
rs114165349	G	0.147891	C	0.053148	-	-	C	0.0930986

rs2374456	G	0.00770514	-	-	G	0.0235779	G	0.0158069
rs738409	G	0.0512185	-	-	G	0.0147054	C	0.0170731
rs6792725	G	0.0189371	-	-	-	-	A	0.0172911
rs7065171	G	0.0179545	-	-	G	0.00634344	-	-
rs11937496	G	0.0070501	-	-	G	0.0070501	-	-
rs4938576	G	0.00717531	-	-	G	0.00717531	-	-
rs57615517	G	0.00804383	-	-	G	0.00804383	-	-
rs696516	G	0.00845398	-	-	G	0.00845398	-	-
rs6939861	G	0.0250047	-	-	G	0.00864192	-	-
rs2287322	G	0.00870892	-	-	G	0.00870892	-	-
rs4782568	G	0.0247859	-	-	G	0.00873274	-	-
rs77741622	G	0.00878066	-	-	G	0.00878066	-	-
rs10202148	G	0.00884922	-	-	G	0.00884922	-	-
rs221584	G	0.00956269	-	-	G	0.00956269	-	-
rs12788072	G	0.00959273	-	-	G	0.00959273	-	-
rs36205397	G	0.00963332	-	-	G	0.00963332	-	-
rs35698268	G	0.00967281	-	-	G	0.00967281	-	-
rs17810415	G	0.00970654	-	-	G	0.00970654	-	-
rs180435	G	0.009709	-	-	G	0.009709	-	-
rs370222	G	0.0100454	-	-	G	0.0100454	-	-
rs61320678	G	0.0100581	-	-	G	0.0100581	-	-
rs520829	G	0.0100721	-	-	G	0.0100721	-	-
rs2723065	G	0.0105151	-	-	G	0.0105151	-	-
rs62314881	G	0.0106611	-	-	G	0.0106611	-	-
rs5752773	G	0.0108792	-	-	G	0.0108792	-	-
rs56271032	G	0.0110385	-	-	G	0.0110385	-	-
rs6471583	G	0.0113891	-	-	G	0.0113891	-	-
rs1408	G	0.0114428	-	-	G	0.0114428	-	-
rs56117787	G	0.0126732	-	-	G	0.0126732	-	-
rs2273991	G	0.0128192	-	-	G	0.0128192	-	-
rs28576256	G	0.0133849	-	-	G	0.0133849	-	-
rs73075656	G	0.0147509	-	-	G	0.0147509	-	-
rs41378347	G	0.0148605	-	-	G	0.0148605	-	-

rs34060476	G	0.0197945	-	-	G	0.0197945	-	-
rs9638084	G	0.00725485	-	-	G	0.0206264	-	-
rs150539196	G	0.021834	-	-	G	0.021834	-	-
rs4453027	G	0.0101731	-	-	G	0.0226255	-	-
rs62162863	G	0.00957424	-	-	G	0.0230675	-	-
rs1870940	G	0.0104433	-	-	G	0.0253915	-	-
rs4245930	G	0.00730443	-	-	G	0.0254406	-	-
rs784420	G	0.0129787	-	-	G	0.0390185	-	-
rs17089026	G	0.0530863	-	-	G	0.0530863	-	-
rs7342537	G	0.0357478	-	-	G	0.106873	-	-
rs62076019	G	0.0156391	-	-	-	-	-	-
rs2540945	G	0.0175945	-	-	-	-	-	-
rs329122	G	0.017745	-	-	-	-	-	-
rs4757142	G	0.0178616	-	-	-	-	-	-
rs12910403	G	0.0182335	-	-	-	-	-	-
rs8107967	G	0.0186513	-	-	-	-	-	-
rs72721770	G	0.0191253	-	-	-	-	-	-
rs3809272	G	0.0195579	-	-	-	-	-	-
rs12787293	G	0.0199201	-	-	-	-	-	-
rs13289095	G	0.0206541	-	-	-	-	-	-
rs2239222	G	0.0206562	-	-	-	-	-	-
rs7182912	G	0.020973	-	-	-	-	-	-
rs1441911	G	0.0215233	-	-	-	-	-	-
rs12445820	G	0.0247469	-	-	-	-	-	-
rs841194	G	0.0271929	-	-	-	-	-	-
rs56853305	G	0.0272089	-	-	-	-	-	-
rs1154401	G	0.028057	-	-	-	-	-	-
rs3768321	G	0.0308558	-	-	-	-	-	-
rs4841133	G	0.0311367	-	-	-	-	-	-
rs55867305	G	0.0326321	-	-	-	-	-	-
rs9970140	G	0.0401518	-	-	-	-	-	-
rs645040	G	0.0432646	-	-	-	-	-	-
rs34851490	G	0.0476696	-	-	-	-	-	-

rs79717793	G	0.0493115	-	-	-	-	-	-
rs28394864	G	0.0529464	-	-	-	-	-	-
rs7015	G	0.0558943	-	-	-	-	-	-
rs145843487	G	0.0755624	-	-	-	-	-	-
rs550628400	G	0.134374	-	-	-	-	-	-
rs79488654	G	0.235323	-	-	-	-	-	-
rs761954625	CTG	0.0317033	-	-	-	-	-	-
rs34632394	CAT	0.00761219	-	-	CAT	0.0166873	-	-
rs11621792	C	0.0176248	T	0.01275	-	-	T	0.0245363
rs202200760	C	0.120055	G	0.0396479	-	-	G	0.0627387
rs2038695	C	0.0209761	C	0.0227854	C	0.00719174	C	0.0113985
rs171021	C	0.0135666	C	0.0127562	C	0.0383767	C	0.0180676
rs12796488	C	0.0412399	C	0.0560936	C	0.0095618	C	0.0248549
rs7183977	C	0.021492	C	0.0188057	C	0.021492	C	0.0285915
rs8111359	C	0.0204451	C	0.0210898	C	0.0597743	C	0.0343229
rs112265145	C	0.0976096	C	0.0854405	C	0.0347973	C	0.0854405
rs17853284	C	0.117717	C	0.111748	C	0.30434	C	0.16988
rs6486542	C	0.0212154	C	0.0261149	-	-	C	0.0158958
rs7912521	C	0.0492378	C	0.061199	-	-	C	0.0306435
rs2668776	C	0.0228009	C	0.0292238	-	-	-	-
rs6258	C	0.717993	-	-	C	0.2178	T	0.554565
rs6020423	C	0.0102645	-	-	C	0.0398225	C	0.0250085
rs6684361	C	0.0247434	-	-	C	0.07582	C	0.0395286
rs74652944	C	0.0658482	-	-	C	0.182556	C	0.106351
rs35008345	C	0.0590811	-	-	C	0.182752	C	0.131046
rs11888201	C	0.00701206	-	-	C	0.00701206	-	-
rs4725944	C	0.00727243	-	-	C	0.00727243	-	-
rs4912377	C	0.00763583	-	-	C	0.00763583	-	-
rs9543012	C	0.00806298	-	-	C	0.00806298	-	-
rs35182096	C	0.023075	-	-	C	0.00810483	-	-
rs13152154	C	0.00830777	-	-	C	0.00830777	-	-
rs157934	C	0.00833858	-	-	C	0.00833858	-	-
rs35222808	C	0.00907747	-	-	C	0.00907747	-	-

rs78851238	C	0.00933861	-	-	C	0.00933861	-	-
rs7686914	C	0.0110552	-	-	C	0.0110552	-	-
rs57323441	C	0.0114104	-	-	C	0.0114104	-	-
rs445	C	0.0118382	-	-	C	0.0118382	-	-
rs10017280	C	0.0122688	-	-	C	0.0122688	-	-
rs2583949	C	0.0123421	-	-	C	0.0123421	-	-
rs9686661	C	0.012459	-	-	C	0.012459	-	-
rs73212896	C	0.0126296	-	-	C	0.0126296	-	-
rs11572082	C	0.0129824	-	-	C	0.0129824	-	-
rs35816571	C	0.0140933	-	-	C	0.0140933	-	-
rs11830764	C	0.0149059	-	-	C	0.0149059	-	-
rs67967246	C	0.0152385	-	-	C	0.0152385	-	-
rs34858588	C	0.0155256	-	-	C	0.0155256	-	-
rs2879910	C	0.00793705	-	-	C	0.0160429	-	-
rs1317701	C	0.0167637	-	-	C	0.0167637	-	-
rs494242	C	0.00804006	-	-	C	0.0168975	-	-
rs9509847	C	0.0200719	-	-	C	0.0200719	-	-
rs9850919	C	0.00692584	-	-	C	0.0201136	-	-
rs12185851	C	0.00874017	-	-	C	0.0214051	-	-
rs55885610	C	0.022606	-	-	C	0.022606	-	-
rs79384925	C	0.0255801	-	-	C	0.0255801	-	-
rs55707100	C	0.110844	-	-	C	0.0269106	-	-
rs62115715	C	0.0295327	-	-	C	0.0295327	-	-
rs12436785	C	0.0105394	-	-	C	0.0300452	-	-
rs1547308	C	0.0123006	-	-	C	0.0322875	-	-
rs1260326	C	0.06159	-	-	C	0.036635	-	-
rs7618363	C	0.0116041	-	-	C	0.0370916	-	-
rs72681869	C	0.103393	-	-	C	0.0419559	-	-
rs10817260	C	0.0137891	-	-	C	0.0429746	-	-
rs4464040	C	0.0144835	-	-	C	0.0445846	-	-
rs535305978	C	0.0453813	-	-	C	0.0453813	-	-
rs36032941	C	0.0225548	-	-	C	0.0628496	-	-
rs2957683	C	0.016475	-	-	-	-	-	-

rs11671304	C	0.0164771	-	-	-	-	-	-
rs3808869	C	0.0166154	-	-	-	-	-	-
rs11096640	C	0.0174617	-	-	-	-	-	-
rs2862954	C	0.0182502	-	-	-	-	-	-
rs12336359	C	0.0186014	-	-	-	-	-	-
rs10028954	C	0.0187935	-	-	-	-	-	-
rs60701	C	0.0188023	-	-	-	-	-	-
rs17408832	C	0.0189949	-	-	-	-	-	-
rs12543287	C	0.019905	-	-	-	-	-	-
rs7844586	C	0.0200617	-	-	-	-	-	-
rs2551641	C	0.0215672	-	-	-	-	-	-
rs650558	C	0.0215805	-	-	-	-	-	-
rs10864086	C	0.02166	-	-	-	-	-	-
rs10750766	C	0.0220518	-	-	-	-	-	-
rs534541609	C	0.0221508	-	-	-	-	-	-
rs7773995	C	0.0235835	-	-	-	-	-	-
rs1203109	C	0.0237647	-	-	-	-	-	-
rs7824394	C	0.0238511	-	-	-	-	-	-
rs7835492	C	0.0245564	-	-	-	-	-	-
rs7143218	C	0.0246549	-	-	-	-	-	-
rs11607114	C	0.0252391	-	-	-	-	-	-
rs55869022	C	0.0267278	-	-	-	-	-	-
rs7735249	C	0.0284808	-	-	-	-	-	-
rs34131245	C	0.0295835	-	-	-	-	-	-
rs62062271	C	0.0305695	-	-	-	-	-	-
rs4525526	C	0.0334533	-	-	-	-	-	-
rs77044968	C	0.0371362	-	-	-	-	-	-
rs35824797	C	0.0440261	-	-	-	-	-	-
rs2012736	C	0.0481941	-	-	-	-	-	-
rs146447930	C	0.0710861	-	-	-	-	-	-
rs17580	A	0.048637	T	0.0270762	-	-	T	0.0401832
rs1799941	A	0.196601	G	0.0339594	A	0.0643261	G	0.0627545
rs13108218	A	0.0348455	G	0.0176657	-	-	G	0.0176657

rs56332871	A	0.0472832	C	0.0181845	A	0.0172791	C	0.0298823
rs56196860	A	0.300859	A	0.321256	C	0.0575327	C	0.047541
rs2764772	A	0.031954	A	0.0370436	A	0.00828619	A	0.0189214
rs113017476	A	0.190415	A	0.182427	A	0.048041	A	0.0874817
rs10279715	A	0.0183219	A	0.0217388	-	-	A	0.014209
rs9986829	A	0.0413601	A	0.0556073	-	-	A	0.0279447
rs40270	A	0.0219284	-	-	-	-	C	0.0189592
rs12683780	A	0.0104924	-	-	A	0.0308941	A	0.0179481
rs35783704	A	0.0200758	-	-	A	0.0200758	A	0.0326591
rs7937758	A	0.00663707	-	-	A	0.00663707	-	-
rs12914034	A	0.00693667	-	-	A	0.00693667	-	-
rs13185520	A	0.0078686	-	-	A	0.0078686	-	-
rs6462989	A	0.00798825	-	-	A	0.00798825	-	-
rs2201003	A	0.00800069	-	-	A	0.00800069	-	-
rs62220604	A	0.00836975	-	-	A	0.00836975	-	-
rs1822246	A	0.0083795	-	-	A	0.0083795	-	-
rs3103310	A	0.00895949	-	-	A	0.00895949	-	-
rs73200740	A	0.00921089	-	-	A	0.00921089	-	-
rs72708239	A	0.00963222	-	-	A	0.00963222	-	-
rs12702516	A	0.0110939	-	-	A	0.0110939	-	-
rs759068	A	0.0114466	-	-	A	0.0114466	-	-
rs267733	A	0.0228514	-	-	A	0.0118795	-	-
rs28495625	A	0.0127398	-	-	A	0.0127398	-	-
rs4804669	A	0.0131493	-	-	A	0.0131493	-	-
rs41264630	A	0.0135205	-	-	A	0.0135205	-	-
rs9913470	A	0.0138148	-	-	A	0.0138148	-	-
rs1681967	A	0.0149523	-	-	A	0.0149523	-	-
rs58839393	A	0.0157925	-	-	A	0.0157925	-	-
rs36182456	A	0.0184446	-	-	A	0.0184446	-	-
rs78248023	A	0.0192124	-	-	A	0.0192124	-	-
rs11191801	A	0.00919391	-	-	A	0.0234295	-	-
rs2903385	A	0.00939408	-	-	A	0.024558	-	-
rs5751229	A	0.0110158	-	-	A	0.0250506	-	-

rs5763800	A	0.0267132	-	-	A	0.0267132	-	-
rs4632729	A	0.0148349	-	-	A	0.02758	-	-
rs11673591	A	0.0123488	-	-	A	0.0284525	-	-
rs3771243	A	0.00901418	-	-	A	0.0287365	-	-
rs28421540	A	0.0108359	-	-	A	0.030872	-	-
rs79391862	A	0.157273	-	-	A	0.0407221	-	-
rs112694713	A	0.0406377	-	-	A	0.106962	-	-
rs138983180	A	0.0635131	-	-	A	0.162237	-	-
rs200647710	A	0.0161679	-	-	-	-	-	-
rs42945	A	0.0162178	-	-	-	-	-	-
rs7828742	A	0.0167984	-	-	-	-	-	-
rs12125882	A	0.0168277	-	-	-	-	-	-
rs12470971	A	0.0170308	-	-	-	-	-	-
rs7997628	A	0.0172862	-	-	-	-	-	-
rs1349359	A	0.0173028	-	-	-	-	-	-
rs9383605	A	0.0190165	-	-	-	-	-	-
rs7166920	A	0.0196875	-	-	-	-	-	-
rs59194935	A	0.0200183	-	-	-	-	-	-
rs10958704	A	0.0200814	-	-	-	-	-	-
rs746303202	A	0.020142	-	-	-	-	-	-
rs45490496	A	0.0207827	-	-	-	-	-	-
rs775181992	A	0.023258	-	-	-	-	-	-
rs12926107	A	0.0243727	-	-	-	-	-	-
rs10832570	A	0.0251653	-	-	-	-	-	-
rs34702488	A	0.0258822	-	-	-	-	-	-
rs2393775	A	0.0263801	-	-	-	-	-	-
rs2306216	A	0.0266929	-	-	-	-	-	-
rs6676846	A	0.0296264	-	-	-	-	-	-
rs11772470	A	0.0301631	-	-	-	-	-	-
rs1624295	A	0.0328259	-	-	-	-	-	-
rs2583948	A	0.0349086	-	-	-	-	-	-
rs7696472	A	0.0382386	-	-	-	-	-	-
rs6750410	A	0.0420059	-	-	-	-	-	-

rs12320328	A	0.0431479	-	-	-	-	-	-
rs73079476	A	0.0544901	-	-	-	-	-	-
rs76767219	A	0.0548531	-	-	-	-	-	-
rs6736913	A	0.0619186	-	-	-	-	-	-
rs112765699	A	0.0747999	-	-	-	-	-	-
rs4826645	-	-	T	0.00947101	-	-	T	0.00947101
rs2504791	-	-	T	0.0110148	-	-	T	0.0110148
rs4655598	-	-	T	0.0111549	-	-	T	0.0111549
rs2781379	-	-	T	0.0118666	-	-	T	0.0118666
rs7496293	-	-	T	0.0233129	-	-	T	0.0121045
rs34192788	-	-	T	0.0198155	-	-	T	0.0130377
rs2081687	-	-	T	0.0131537	-	-	T	0.0131537
rs6854749	-	-	T	0.0144127	-	-	T	0.0144127
rs6502026	-	-	T	0.0149619	-	-	T	0.0149619
rs2248979	-	-	T	0.0159999	-	-	T	0.0159999
rs10978435	-	-	T	0.0175351	-	-	T	0.0175351
rs6950023	-	-	T	0.0180401	-	-	T	0.0180401
rs6009583	-	-	T	0.018393	-	-	T	0.018393
rs11078597	-	-	T	0.0150877	-	-	T	0.0192985
rs2392239	-	-	T	0.0193946	-	-	T	0.0193946
rs41306249	-	-	T	0.0417541	-	-	T	0.0221593
rs138271349	-	-	T	0.0284812	-	-	T	0.0227996
rs41264945	-	-	T	0.0244992	-	-	T	0.0244992
rs76006132	-	-	T	0.0322757	-	-	T	0.0322757
rs8178824	-	-	T	0.04606	-	-	T	0.0577784
rs148664256	-	-	T	0.0755948	-	-	T	0.0755948
rs76895963	-	-	T	0.065956	-	-	T	0.0875478
rs545206972	-	-	T	0.251792	-	-	T	0.427953
rs1454836	-	-	T	0.0166598	-	-	-	-
rs7454964	-	-	T	0.0171983	-	-	-	-
rs4483209	-	-	T	0.0173447	-	-	-	-
rs6729954	-	-	T	0.0189552	-	-	-	-
rs1178947	-	-	T	0.0238711	-	-	-	-

rs9824196	-	-	T	0.025986	-	-	-	-
rs6718154	-	-	T	0.0322455	-	-	-	-
rs2035837	-	-	T	0.07286	-	-	-	-
rs116923389	-	-	T	0.0979365	-	-	-	-
rs11707420	-	-	G	0.0116799	-	-	G	0.0116799
rs1318995	-	-	G	0.0118184	-	-	G	0.0118184
rs10745954	-	-	G	0.0129654	-	-	G	0.0129654
rs998616	-	-	G	0.0132641	-	-	G	0.0132641
rs2350892	-	-	G	0.014464	-	-	G	0.014464
rs7248104	-	-	G	0.0127354	-	-	G	0.0156963
rs4565819	-	-	G	0.0158151	-	-	G	0.0158151
rs7610095	-	-	G	0.0160018	-	-	G	0.0160018
rs13261573	-	-	G	0.0161763	-	-	G	0.0161763
rs3180887	-	-	G	0.016323	-	-	G	0.016323
rs11155076	-	-	G	0.0176973	-	-	G	0.0176973
rs72834698	-	-	G	0.0177982	-	-	G	0.0177982
rs1937883	-	-	G	0.0182063	-	-	G	0.0182063
rs13028479	-	-	G	0.0308994	-	-	G	0.0184182
rs10212007	-	-	G	0.01963	-	-	G	0.01963
rs112295236	-	-	G	0.0313604	-	-	G	0.0313604
rs5915287	-	-	G	0.0123973	-	-	-	-
rs10218066	-	-	G	0.014648	-	-	-	-
rs6616174	-	-	G	0.0157969	-	-	-	-
rs829593	-	-	G	0.0177819	-	-	-	-
rs1112195	-	-	G	0.0182475	-	-	-	-
rs503542	-	-	G	0.0183001	-	-	-	-
rs1034948	-	-	G	0.0183286	-	-	-	-
rs2438086	-	-	G	0.0218279	-	-	-	-
rs3821866	-	-	G	0.0221282	-	-	-	-
rs4872310	-	-	G	0.0231059	-	-	-	-
rs62041532	-	-	G	0.0233361	-	-	-	-
rs12907068	-	-	G	0.0234292	-	-	-	-
rs4503095	-	-	G	0.0300118	-	-	-	-

rs7265992	-	-	G	0.0317217	-	-	-	-
rs13065463	-	-	G	0.0320519	-	-	-	-
rs4562360	-	-	G	0.0323526	-	-	-	-
rs2631864	-	-	G	0.0331054	-	-	-	-
rs79310511	-	-	G	0.0433605	-	-	-	-
rs5913997	-	-	G	0.0526047	-	-	-	-
rs11751920	-	-	G	0.0605865	-	-	-	-
rs146225865	-	-	G	0.0943468	-	-	-	-
rs190930099	-	-	G	0.134766	-	-	-	-
rs4149056	-	-	C	0.0231508	C	0.0289515	C	0.0425623
rs5970021	-	-	C	0.0111221	-	-	C	0.0111221
rs2312064	-	-	C	0.0111847	-	-	C	0.0111847
rs1104608	-	-	C	0.0119033	-	-	C	0.0119033
rs1851292	-	-	C	0.012152	-	-	C	0.012152
rs7395670	-	-	C	0.0122007	-	-	C	0.0122007
rs12713012	-	-	C	0.0123839	-	-	C	0.0123839
rs7626226	-	-	C	0.0124214	-	-	C	0.0124214
rs7256062	-	-	C	0.0127754	-	-	C	0.0127754
rs35319517	-	-	C	0.0144524	-	-	C	0.0144524
rs2587507	-	-	C	0.0117181	-	-	C	0.0144984
rs71529289	-	-	C	0.0359428	-	-	C	0.017019
rs5905042	-	-	C	0.0228475	-	-	C	0.0174486
rs9322822	-	-	C	0.0487092	-	-	C	0.023054
rs1264332	-	-	C	0.0233666	-	-	C	0.0233666
rs17703883	-	-	C	0.0442784	-	-	C	0.0243257
rs799158	-	-	C	0.0277953	-	-	C	0.0277953
rs629042	-	-	C	0.0173631	-	-	C	0.0284066
rs11655191	-	-	C	0.0391797	-	-	C	0.0391797
rs147676232	-	-	C	0.0674853	-	-	C	0.0459525
rs188539658	-	-	C	0.0532722	-	-	C	0.0532722
rs243466	-	-	C	0.0130627	-	-	-	-
rs10510939	-	-	C	0.0173999	-	-	-	-
rs4274916	-	-	C	0.0174588	-	-	-	-

rs2327121	-	-	C	0.0181409	-	-	-	-
rs72664935	-	-	C	0.0186769	-	-	-	-
rs2961853	-	-	C	0.0191152	-	-	-	-
rs6521	-	-	C	0.0193524	-	-	-	-
rs2184968	-	-	C	0.0202431	-	-	-	-
rs745486	-	-	C	0.0213054	-	-	-	-
rs61932784	-	-	C	0.022236	-	-	-	-
rs58879558	-	-	C	0.0225798	-	-	-	-
rs8076703	-	-	C	0.0238709	-	-	-	-
rs55765314	-	-	C	0.0247992	-	-	-	-
rs12410063	-	-	C	0.0256367	-	-	-	-
rs1272131	-	-	C	0.0267642	-	-	-	-
rs72774885	-	-	C	0.0268849	-	-	-	-
rs912202	-	-	C	0.0401986	-	-	-	-
rs10137488	-	-	C	0.0532906	-	-	-	-
rs140498714	-	-	C	0.0544025	-	-	-	-
rs73221538	-	-	C	0.055959	-	-	-	-
rs17254118	-	-	C	0.109118	-	-	-	-
rs111386834	-	-	C	0.1322	-	-	-	-
rs61237993	-	-	A	0.0180937	A	0.0399573	A	0.0294017
rs1180894	-	-	A	0.0111935	-	-	A	0.0111935
rs11588341	-	-	A	0.0120369	-	-	A	0.0120369
rs4828043	-	-	A	0.0126916	-	-	A	0.0126916
rs12929087	-	-	A	0.0129587	-	-	A	0.0129587
rs11919691	-	-	A	0.0132684	-	-	A	0.0132684
rs6142206	-	-	A	0.0133602	-	-	A	0.0133602
rs6912283	-	-	A	0.0138991	-	-	A	0.0138991
rs4237037	-	-	A	0.0164615	-	-	A	0.0164615
rs11240695	-	-	A	0.0183046	-	-	A	0.0183046
rs11510917	-	-	A	0.0183953	-	-	A	0.0183953
rs7780066	-	-	A	0.0134951	-	-	A	0.0227824
rs794614	-	-	A	0.0229194	-	-	A	0.0229194
rs73969326	-	-	A	0.0230056	-	-	A	0.0230056

rs73241342	-	-	A	0.0286638	-	-	A	0.0286638
rs9987289	-	-	A	0.0252994	-	-	A	0.0336644
rs7872329	-	-	A	0.0174383	-	-	-	-
rs757309	-	-	A	0.0178789	-	-	-	-
rs13835	-	-	A	0.0189802	-	-	-	-
rs10738700	-	-	A	0.0204816	-	-	-	-
rs4919686	-	-	A	0.0234107	-	-	-	-
rs71519251	-	-	A	0.0240397	-	-	-	-
rs3733897	-	-	A	0.0250318	-	-	-	-
rs204995	-	-	A	0.0272612	-	-	-	-
rs8061590	-	-	A	0.0288317	-	-	-	-
rs950716	-	-	A	0.0336833	-	-	-	-
rs573833	-	-	-	-	-	-	T	0.0148264
rs34341	-	-	-	-	-	-	T	0.0149201
rs2282248	-	-	-	-	-	-	T	0.0150665
rs1515098	-	-	-	-	-	-	T	0.0153085
rs13092573	-	-	-	-	-	-	T	0.0155943
rs6538437	-	-	-	-	-	-	T	0.0161517
rs351370	-	-	-	-	-	-	T	0.0162176
rs6486122	-	-	-	-	-	-	T	0.0167011
rs12645584	-	-	-	-	-	-	T	0.0169337
rs4368453	-	-	-	-	-	-	T	0.0170734
rs62144584	-	-	-	-	-	-	T	0.0188548
rs12543598	-	-	-	-	-	-	T	0.0199058
rs168189	-	-	-	-	-	-	T	0.0202832
rs6772177	-	-	-	-	-	-	T	0.0203877
rs62396733	-	-	-	-	-	-	T	0.0213341
rs17287714	-	-	-	-	-	-	T	0.0220873
rs7089122	-	-	-	-	-	-	T	0.024365
rs2391139	-	-	-	-	-	-	T	0.0246704
rs4690098	-	-	-	-	-	-	T	0.0266003
rs7078330	-	-	-	-	-	-	T	0.0312818
rs2152318	-	-	-	-	-	-	T	0.0317947

rs850294	-	-	-	-	-	-	T	0.0337033
rs764029425	-	-	-	-	-	-	T	0.0364696
rs687339	-	-	-	-	-	-	T	0.0395716
rs149624078	-	-	-	-	-	-	T	0.0791193
rs727428	-	-	-	-	-	-	T	0.0947823
rs11235688	-	-	-	-	G	0.0222542	G	0.0164676
rs7633673	-	-	-	-	G	0.0255585	G	0.018352
rs6008259	-	-	-	-	G	0.0338188	G	0.0208006
rs112635299	-	-	-	-	G	0.0621054	G	0.102348
rs381194	-	-	-	-	-	-	G	0.0127199
rs12135478	-	-	-	-	-	-	G	0.0150944
rs62223042	-	-	-	-	-	-	G	0.0153338
rs4135247	-	-	-	-	-	-	G	0.0153765
rs13072623	-	-	-	-	-	-	G	0.0153889
rs7418101	-	-	-	-	-	-	G	0.0155018
rs13430258	-	-	-	-	-	-	G	0.0155063
rs17128091	-	-	-	-	-	-	G	0.0162945
rs13030651	-	-	-	-	-	-	G	0.0165893
rs10504255	-	-	-	-	-	-	G	0.0173977
rs1214759	-	-	-	-	-	-	G	0.018082
rs3814707	-	-	-	-	-	-	G	0.0195128
rs71397837	-	-	-	-	-	-	G	0.0196459
rs56205943	-	-	-	-	-	-	G	0.0209185
rs879619	-	-	-	-	-	-	G	0.0210598
rs11683361	-	-	-	-	-	-	G	0.0222847
rs3732218	-	-	-	-	-	-	G	0.0252069
rs77031559	-	-	-	-	-	-	G	0.0273345
rs12189146	-	-	-	-	-	-	G	0.0320071
rs11047261	-	-	-	-	-	-	G	0.0366187
rs191780890	-	-	-	-	-	-	G	0.0660169
rs11733695	-	-	-	-	-	-	G	0.0811767
rs17245822	-	-	-	-	C	0.0283937	C	0.013936
rs1119208	-	-	-	-	C	0.0304668	C	0.0174527

rs13153019	-	-	-	-	C	0.0242892	C	0.0177496
rs11031005	-	-	-	-	C	0.0325862	C	0.0229697
rs62231822	-	-	-	-	C	0.0489104	C	0.0288713
rs34269793	-	-	-	-	C	0.0644978	C	0.033339
rs34931250	-	-	-	-	C	0.0544774	C	0.0399468
rs58072681	-	-	-	-	C	0.108156	C	0.0610878
rs1432679	-	-	-	-	-	-	C	0.0133517
rs11784903	-	-	-	-	-	-	C	0.0135555
rs4869893	-	-	-	-	-	-	C	0.0155066
rs2954111	-	-	-	-	-	-	C	0.0166682
rs388430	-	-	-	-	-	-	C	0.0167293
rs9636441	-	-	-	-	-	-	C	0.0167895
rs13020003	-	-	-	-	-	-	C	0.0170182
rs8046391	-	-	-	-	-	-	C	0.0170855
rs4712976	-	-	-	-	-	-	C	0.0186329
rs10851395	-	-	-	-	-	-	C	0.0189317
rs6129778	-	-	-	-	-	-	C	0.0196229
rs34255979	-	-	-	-	-	-	C	0.0227729
rs9527	-	-	-	-	-	-	C	0.0230347
rs1989147	-	-	-	-	-	-	C	0.0235512
rs5970442	-	-	-	-	-	-	C	0.0267306
rs199816133	-	-	-	-	-	-	C	0.0286444
rs7239564	-	-	-	-	-	-	C	0.0307663
rs61856128	-	-	-	-	-	-	C	0.0346
rs1688043	-	-	-	-	-	-	C	0.0406564
rs113172275	-	-	-	-	-	-	C	0.0491508
rs11653686	-	-	-	-	-	-	C	0.0656834
rs746258907	-	-	-	-	-	-	AG	0.0155878
rs12564492	-	-	-	-	A	0.0195027	A	0.0153482
rs117327231	-	-	-	-	A	0.173128	A	0.0971595
rs10757893	-	-	-	-	-	-	A	0.0137428
rs2397112	-	-	-	-	-	-	A	0.0161299
rs2266782	-	-	-	-	-	-	A	0.0161462

rs7301634	-	-	-	-	-	-	A	0.0191215
rs9399469	-	-	-	-	-	-	A	0.0198083
rs6788984	-	-	-	-	-	-	A	0.0200685
rs73350117	-	-	-	-	-	-	A	0.0202695
rs1640272	-	-	-	-	-	-	A	0.0211934
rs9379084	-	-	-	-	-	-	A	0.0224985
rs10778215	-	-	-	-	-	-	A	0.024324
rs62263023	-	-	-	-	-	-	A	0.0281185
rs78058190	-	-	-	-	-	-	A	0.0408659
rs114303452	-	-	-	-	-	-	A	0.0918637
rs181255261	-	-	-	-	-	-	A	0.116295
rs6460528	-	-	-	-	T	0.0152877	-	-
rs2608652	-	-	-	-	T	0.0154434	-	-
rs6904345	-	-	-	-	T	0.0165472	-	-
rs11948639	-	-	-	-	T	0.017095	-	-
rs35427	-	-	-	-	T	0.0171038	-	-
rs167096	-	-	-	-	T	0.017315	-	-
rs9599996	-	-	-	-	T	0.0174262	-	-
rs3032555	-	-	-	-	T	0.0175831	-	-
rs7530117	-	-	-	-	T	0.0177946	-	-
rs2147419	-	-	-	-	T	0.018171	-	-
rs7977247	-	-	-	-	T	0.0182888	-	-
rs9898480	-	-	-	-	T	0.0182946	-	-
rs72738949	-	-	-	-	T	0.0188926	-	-
rs11697333	-	-	-	-	T	0.0191659	-	-
rs1242518	-	-	-	-	T	0.0204549	-	-
rs10865479	-	-	-	-	T	0.0221171	-	-
rs1660322	-	-	-	-	T	0.0233139	-	-
rs76830943	-	-	-	-	T	0.0242358	-	-
rs17043570	-	-	-	-	T	0.0254051	-	-
rs13184921	-	-	-	-	T	0.0254693	-	-
rs2113944	-	-	-	-	T	0.0271852	-	-
rs12078363	-	-	-	-	T	0.0276185	-	-

rs437115	-	-	-	-	T	0.0278697	-	-
rs36088520	-	-	-	-	T	0.0283274	-	-
rs75848431	-	-	-	-	T	0.0316829	-	-
rs62059839	-	-	-	-	T	0.0324826	-	-
rs10168169	-	-	-	-	T	0.0333376	-	-
rs7519368	-	-	-	-	T	0.0334167	-	-
rs58723250	-	-	-	-	T	0.0351228	-	-
rs4736359	-	-	-	-	T	0.0370075	-	-
rs67596711	-	-	-	-	T	0.0386841	-	-
rs75287599	-	-	-	-	T	0.0432724	-	-
rs17201704	-	-	-	-	T	0.0433007	-	-
rs117913411	-	-	-	-	T	0.0481096	-	-
rs1872930	-	-	-	-	T	0.0514302	-	-
rs9506725	-	-	-	-	T	0.0523465	-	-
rs11638521	-	-	-	-	T	0.0639626	-	-
rs267190	-	-	-	-	G	0.0152314	-	-
rs28612846	-	-	-	-	G	0.0155678	-	-
rs505237	-	-	-	-	G	0.0157305	-	-
rs837493	-	-	-	-	G	0.0158241	-	-
rs13094915	-	-	-	-	G	0.0163011	-	-
rs3776299	-	-	-	-	G	0.0170666	-	-
rs2270445	-	-	-	-	G	0.0171622	-	-
rs2344744	-	-	-	-	G	0.0178199	-	-
rs10108398	-	-	-	-	G	0.0180668	-	-
rs7256920	-	-	-	-	G	0.0188528	-	-
rs12837203	-	-	-	-	G	0.0192885	-	-
rs4294422	-	-	-	-	G	0.0193786	-	-
rs1169289	-	-	-	-	G	0.0194662	-	-
rs35801460	-	-	-	-	G	0.0204154	-	-
rs17764067	-	-	-	-	G	0.0207512	-	-
rs12708515	-	-	-	-	G	0.0213711	-	-
rs10501081	-	-	-	-	G	0.0216363	-	-
rs17362923	-	-	-	-	G	0.0233287	-	-

rs4067	-	-	-	-	G	0.0242583	-	-
rs72731535	-	-	-	-	G	0.0253928	-	-
rs440150	-	-	-	-	G	0.0256632	-	-
rs56109436	-	-	-	-	G	0.0258972	-	-
rs9687846	-	-	-	-	G	0.0263617	-	-
rs12628709	-	-	-	-	G	0.0264022	-	-
rs10799713	-	-	-	-	G	0.0270994	-	-
rs312023	-	-	-	-	G	0.0279464	-	-
rs1214761	-	-	-	-	G	0.0311146	-	-
rs59741822	-	-	-	-	G	0.0329755	-	-
rs1883711	-	-	-	-	G	0.0499885	-	-
rs59397130	-	-	-	-	G	0.056163	-	-
rs749512116	-	-	-	-	CAGAT	0.0328564	-	-
rs11427441	-	-	-	-	CA	0.0200041	-	-
rs10821415	-	-	-	-	C	0.014599	-	-
rs873779	-	-	-	-	C	0.0164017	-	-
rs2062479	-	-	-	-	C	0.016408	-	-
rs10910476	-	-	-	-	C	0.0168419	-	-
rs35199395	-	-	-	-	C	0.0170314	-	-
rs61987429	-	-	-	-	C	0.0174663	-	-
rs232159	-	-	-	-	C	0.0175235	-	-
rs674486	-	-	-	-	C	0.0176204	-	-
rs1009360	-	-	-	-	C	0.0179414	-	-
rs7529520	-	-	-	-	C	0.0183162	-	-
rs487624	-	-	-	-	C	0.0193197	-	-
rs6100174	-	-	-	-	C	0.0204027	-	-
rs9611014	-	-	-	-	C	0.0231561	-	-
rs6997799	-	-	-	-	C	0.0235558	-	-
rs3136354	-	-	-	-	C	0.0236592	-	-
rs2186945	-	-	-	-	C	0.0248856	-	-
rs2824138	-	-	-	-	C	0.0259618	-	-
rs61661087	-	-	-	-	C	0.0282982	-	-
rs8044588	-	-	-	-	C	0.0284528	-	-

rs2473140	-	-	-	-	C	0.0292103	-	-
rs111328885	-	-	-	-	C	0.0325591	-	-
rs4804181	-	-	-	-	C	0.0368786	-	-
rs11191421	-	-	-	-	C	0.0374361	-	-
rs7575635	-	-	-	-	C	0.037891	-	-
rs12900736	-	-	-	-	C	0.0397313	-	-
rs1229984	-	-	-	-	C	0.0489634	-	-
rs1032388	-	-	-	-	C	0.0543709	-	-
rs4820829	-	-	-	-	C	0.0575622	-	-
rs11778724	-	-	-	-	C	0.070201	-	-
rs184265581	-	-	-	-	C	0.132322	-	-
rs12977787	-	-	-	-	A	0.0152523	-	-
rs4943729	-	-	-	-	A	0.0154285	-	-
rs2074585	-	-	-	-	A	0.0160303	-	-
rs34163044	-	-	-	-	A	0.0167088	-	-
rs881613	-	-	-	-	A	0.0171593	-	-
rs9832502	-	-	-	-	A	0.017474	-	-
rs6127099	-	-	-	-	A	0.0177481	-	-
rs4586943	-	-	-	-	A	0.0184078	-	-
rs287884	-	-	-	-	A	0.0186304	-	-
rs11024458	-	-	-	-	A	0.0188637	-	-
rs7573187	-	-	-	-	A	0.0189785	-	-
rs3849653	-	-	-	-	A	0.0191787	-	-
rs1278526	-	-	-	-	A	0.0193434	-	-
rs17053931	-	-	-	-	A	0.02055	-	-
rs11892043	-	-	-	-	A	0.0215553	-	-
rs10147094	-	-	-	-	A	0.0219977	-	-
rs8184986	-	-	-	-	A	0.023426	-	-
rs9319895	-	-	-	-	A	0.0234581	-	-
rs1314911	-	-	-	-	A	0.0258707	-	-
rs13269725	-	-	-	-	A	0.0300107	-	-
rs11782259	-	-	-	-	A	0.0307852	-	-
rs72693130	-	-	-	-	A	0.0311903	-	-

rs11125180	-	-	-	-	A	0.0325068	-	-
rs76299412	-	-	-	-	A	0.0349073	-	-
rs75217853	-	-	-	-	A	0.0350023	-	-
rs9552597	-	-	-	-	A	0.0354372	-	-
rs112367565	-	-	-	-	A	0.0491114	-	-
rs62621812	-	-	-	-	A	0.0533948	-	-
rs11774829	-	-	-	-	A	0.056025	-	-
rs187370584	-	-	-	-	A	0.0662026	-	-
rs1939769	-	-	-	-	A	0.0833288	-	-
rs34557412	-	-	-	-	A	0.106892	-	-
